# Supplementary material for: Does Endometriosis Influence the Embryo Quality and/or Development? Insights from a Large Retrospective Matched Cohort Study
Source: Diagnostics (Basel). 2020 Feb 3;10(2):83. doi: 10.3390/diagnostics10020083 (PMC7168899; doi:10.3390/diagnostics10020083)
Supplement: Supplementary file 1 [file diagnostics-10-00083-s001.pdf]

Table S1. Detailed characteristics of the cycles.

	**Controls**	**Endometriosis**
Total number of cycles	851	429
Cycles without mature/fertilized oocytes*	133 (15.6)	61 (14.2)
Cycles without cleavage of fertilized oocytes*	15 (1.8)	10 (2.3)
Cycles with cleavage stage embryos	703 (82.6)	358 (83.4)
Cleavage stage embryos	2483	1314
Cycles with day 3 transfer*	396 (46.5)	192 (44.8)
Cycles with day 3 freeze-all*	31 (3.6)	24 (5.6)
Cycles without transferred or frozen cleavage stage embryos	276 (32.4)	142 (33.1)
Blastocyst stage embryos	770	382
Cycles with day 5 transfer*	120 (14.1)	61 (14.2)
Cycles with blastocyst stage freeze-all*	114 (13.4)	58 (13.5)
Cycles without blastocyst formation*	42 (4.9)	22 (5.1)

*Data were summarized in a contingency table whith 2 groups and 7 categories, *p* value for chi square test equal to 0.73.

Table S2. Frozen embryo transfer details and ongoing pregnancy rate.

	**Controls *n* = 134**	**Endometriosis *n* = 75**	***p*-Value**
Number of transferred embryos, mean ± SD	1.2 ± 0.5	1.3 ± 0.5	0.53
Day 3 transfers	1.3 ± 0.5	1.5 ± 0.6	0.36
Day 5 transfers	1.2 ± 0.4	1.2 ± 0.4	0.88
Number of transfers (%)			
Day 3 transfers	31 (23.1)	17 (22.7)	0.94
Day 5 transfers	103 (76.9)	58 (77.3)	
Ongoing pregnancy rate (95% CI)			
All transfers	29.9 (22.8–38.1)	21.3 (13.6–31.9)	0.24
Adjusted Odds Ratios for ongoing pregnancy rate, (95% CI)			
All transfers*	-	0.44 (0.20–0.93)	0.03

*Adjusted for age, BMI, semen parameters, percentage of mature oocytes, day of the transfer and number of transferred embryos.

Table S3. Causes of infertility in the study population.

	**Controls *n* = 851**	**Endometriosis *n* = 429**
Endometriosis	0 (0.0)	429 (100)
Idiopathic	268 (31.5)	0 (0.0)
Poor ovarian reserve*	324 (38.1)	0(0.0)
Male Infertility**	281 (33.0)	88 (20.5)
Tubal factor	69 (8.1)	28 (6.5)
PGT-M	20 (2.4)	2 (0.5)

* Defined accordingly to Bologna criteria [*Ferraretti et al., 2011*]. ** Total motile sperm count < 5 x 10^6 spermatozoa. PGT-M: Preimplantation genetic testing for monogenic/single gene diseases.

© 2020 by the authors. Submitted for possible open access publication under the terms and conditions of the Creative Commons Attribution (CC BY) license (http://creativecommons.org/licenses/by/4.0/).
